# Evaluation of Clinical and Immunological Characteristics of Children with Common Variable Immunodeficiency

**DOI:** 10.1155/2018/3527480

**Published:** 2018-04-23

**Authors:** Gülsüm Alkan, Sevgi Keles, İsmail Reisli

**Affiliations:** ^1^Department of Pediatric Infectious Diseases, Selcuk University Faculty of Medicine, Konya, Turkey; ^2^Department of Pediatric Allergy and Immunology, Necmettin Erbakan University Meram Faculty of Medicine, Konya, Turkey

## Abstract

**Background:**

Common variable immunodeficiency (CVID) is a primary immunodeficiency disorder (PID) that typically presents with hypogammaglobulinemia and impaired antibody production.

**Objectives:**

This study aimed to promote the awareness of CVID, whose clinical spectrum is quite broad.

**Methods:**

The demographic, clinical, and laboratory characteristics of 12 children (seven males and five females) with CVID were analyzed retrospectively. The patients were diagnosed using the diagnostic criteria of the European Society for Primary Immunodeficiencies.

**Results:**

The median disease onset age was 7.2 ± 4.1 years, and the mean diagnosis age was 11.6 ± 3.7 years. The diagnosis delay was 4.3 ± 2.6 years, and the parental consanguinity rate was 75%. Most patients presented with recurrent infections, including upper respiratory tract infections (*n* = 8), lower respiratory tract infections (*n* = 9), and gastroenteritis (*n* = 5). In addition, growth retardation (*n* = 9) and bronchiectasis (*n* = 5) were common comorbidities. Two patients presented with autoimmune thrombocytopenia and anemia, and one patient exhibited lung empyema. All the patients had immunoglobulin G deficiencies.

**Conclusion:**

CVID is a heterogeneous disease, so the diagnosis is frequently delayed. In the CVID patients with pulmonary complications, relationships were seen with the diagnosis delay, symptom onset age, and lung infection prevalence. Overall, the early diagnosis and treatment of PIDs can preclude life-threatening complications.

## 1. Introduction

Primary immunodeficiency disorders (PIDs) occur due to defects in the development or function of the innate (macrophage, neutrophil, dendritic cell, and complement system) or adaptive (B and T lymphocytes) immune system. Humoral immunodeficiency refers to diseases resulting from impaired antibody production due to a molecular defect intrinsic to the B cells or an interaction failure between the B and T cells [1]. Hypogammaglobulinemia may be primary or secondary, and the primary immunodeficiencies associated with immunoglobulin disorders include selective immunoglobulin (Ig) A deficiency, transient hypogammaglobulinemia of infancy, X-linked agammaglobulinemia (XLA), combined immunodeficiency (CID), common variable immunodeficiency (CVID), and hyper-IgM syndrome. Secondary hypogammaglobulinemia may occur in a wide range of conditions, including nephrotic syndrome, protein-losing enteropathy, chronic kidney disease, and severe malnutrition. XLA is an inherited immunodeficiency disorder characterized by the absence of mature B cells, resulting in a severe antibody deficiency and recurrent infections. Clinically, CVID patients may present similarly to patients with XLA. Certain atypical XLA varieties with a delayed presentation are known to mimic CVID. These are characterized by the presence of B cells and antibody production, albeit in low numbers [2].

CVID is the most frequently occurring PID, and it often presents with hypogammaglobulinemia, recurrent infections, and a poor vaccination response. CVID can present at any age, but it most frequently appears in childhood and early adulthood. The estimated CVID incidence in the general population is between 1 in 10,000 and 1 in 50,000. CVID patients are more prone to recurrent respiratory and gastrointestinal tract infections, and they comprise a large and heterogeneous group of patients. Enteropathy, granulomatous organ infiltrates, malignancy, and inflammatory and autoimmune conditions are also prevalent among these patients. XLA, transient hypogammaglobulinemia, hyperimmunoglobulin M syndromes, and CID disorders should be considered in the differential diagnosis. CVID usually presents in childhood with recurrent sinopulmonary infections, and respiratory morbidity is common, ranging from recurrent suppurative bronchitis to bronchiectasis [3, 4].

Approximately 20–30% of CVID patients develop autoimmune disorders, and this may be the first manifestation of CVID in patients who have no remarkable history of recurrent or severe infections. Many forms of autoimmunity have been described in CVID cases, for example, juvenile rheumatoid arthritis (JRA), systemic lupus erythematosus (SLE), pernicious anemia, autoimmune thyroiditis, alopecia areata, and vitiligo. The most commonly reported types are idiopathic thrombocytopenic purpura (ITP) and autoimmune hemolytic anemia (AIHA) [5].

CVID is characterized by low levels of most or all of the immunoglobulin classes, as well as a lack of B lymphocytes or plasma cells that are capable of producing antibodies. Some patients lack T-helper lymphocytes, which are essential for normal antibody responses. These abnormalities reflect the heterogeneous nature of CVID, and they support the concept that more than one gene is probably responsible for the immune abnormalities in CVID. Although researchers have attempted to determine the genetic variations associated with CVID, over 90% of these cases have not yet been defined on a molecular basis. Sporadic, autosomal-dominant, and autosomal recessive forms have been reported. A heterogeneity of diseases is related to several variations in the genetic background, previously reported as variations in the major histocompatibility complex (MHC) locus genes. Defects in the transmembrane activator and calcium-modulator and cyclophilin ligand interactor (TACI), inducible costimulator (ICOS), B-cell-activating factor receptor (BAFF-R), and nuclear factor kappa B subunit 2 (NFKB2) genes have been identified in some patients with CVID [1, 6, 7]. The use of intravenous gamma globulin has significantly reduced the frequency and severity of infections in CVID patients.

Such a wide spectrum of clinical presentations may cause diagnosis delays. In order to increase awareness and improve CVID diagnoses, we retrospectively reviewed CVID cases with regard to their clinical and laboratory features.

## 2. Materials and Methods

The records of 12 CVID patients who were followed up between 2001 and 2012 at the Meram Medical Faculty of Pediatric Immunology and the Allergic Diseases Clinic at the Necmettin Erbakan University in Konya, Turkey, were evaluated. These patients had been diagnosed with CVID as per the criteria of the European Society for Primary Immunodeficiencies (ESID). We included patients with a marked IgG level decrease (i.e., more than two standard deviations below the mean for one's age) and a marked decrease in the level of at least one of the IgM or IgA isotypes. Additionally, all the included patients fulfilled the following inclusion criteria: immunodeficiency onset at greater than two years of age, absence of isohemagglutinins, and absence of defined causes of hypogammaglobulinemia. Those patients with profound T-cell deficiencies were excluded (CD4 number/microliter: 2–6 years <300, 6–12 years <250, and >12 years <200; % naïve CD4: 2–6 years <25%, 6–16 years <20%, and >16 years 10%). All the patients were evaluated for CID using an exome sequencing analysis (ADA, AK2, GATA2, CD19, RAG1, RAG2, Artemis, BTK, BLNK, PI3K, Ig *α*, and Ig *β*), and those patients with CID were excluded. In addition, secondary hypogammaglobulinemia cases (nephrotic syndrome, protein-losing enteropathy, chronic kidney disease, and severe malnutrition) were excluded.

The following information was collected for each CVID patient: symptom onset age, diagnosis age, admission complaints, parental consanguinity, family history of immunodeficiency, clinical status, physical examination, high-resolution computed tomography (HRCT) findings, and the presence of pulmonary, extrapulmonary, or autoimmune disease. The infection frequency before and after the intravenous immunoglobulin (IVIG) treatment, as well as any supportive therapies used with the IVIG treatment, was also evaluated. The serum immunoglobulin levels were measured nephelometrically. It was considered to be normal for the isohemagglutinin titres to be 1/10 and the anti-hepatitis B (HB) level to be >10 mIU/ml. The lymphocyte subsets from the peripheral blood samples were analyzed using fluorescence-activated cell sorting, as follows: total T cells (CD3+), helper T cells (CD3+ and CD4+), cytotoxic T cells (CD3+ and CD8+), and B cells (CD19+).

The study protocol was reviewed and approved by the appropriate local Ethics and Research Committees (Necmettin Erbakan University Meram Faculty of Medicine).

### 2.1. Statistical Analysis

All the statistical analyses were performed using the Statistical Package for the Social Sciences version 16 (SPSS Inc., Chicago, IL, USA). The data were described in terms of the frequency, median, and mean. The differences among the patient groups were assessed using McNemar's test for the categorical variables, and the statistical significance was set at *p* < 0.05. A nonparametric test (Mann–Whitney *U*) was performed to compare the quantitative variables.

## 3. Results

### 3.1. Demographics

The study population was comprised of 12 children (seven males and five females) whose median age at disease onset was 7.2 ± 4.1 years. Their median age at diagnosis was 11.6 ± 3.7 years, and the diagnosis delay was 4.3 ± 2.6 years. The patient symptoms frequently started before 10 years of age (*n* = 9). There were no significant gender-based differences among the patients in terms of the demographic data (*p* > 0.05). The parental consanguinity rate was 75%, and the PID family history rate was 50% (Table 1).

### 3.2. Physical Examination and Growth

Growth retardation was the most frequent comorbidity (*n* = 9) among the patients. In terms of the pathological physical examination findings, the patients presented with a cervical lymph node (*n* = 1), hepatosplenomegaly (*n* = 4), and verruca (*n* = 5). Hepatosplenomegaly was significantly more common among the autoimmune patients, and crackles were the most common lung examination finding (Table 2).

### 3.3. Infections

Mostly, the patients were referred to our hospital for recurrent upper respiratory tract infections (URIs, *n* = 8), recurrent lower respiratory tract infections (LRIs, *n* = 9), and recurrent gastroenteritis (*n* = 5). Otitis media and sinusitis were the most common URIs. The other 3 patients presented without any frequent infections. Two patients presenting with autoimmune thrombocytopenia and anemia were diagnosed with Evans syndrome by the pediatric hematology department. The other patient presented with lung empyema without a recurrent infection. During the follow-up, three patients were diagnosed with tuberculosis based on the clinical findings and radiology.

### 3.4. Allergies and Asthma

Before being diagnosed with CVID, eight patients were diagnosed with asthma and used corticosteroid inhalers. Only one patient (patient 3) had allergic rhinitis, and the results of her skin-prick test were positive for grass. All the other patients' specific IgE levels or skin-prick test results were normal.

### 3.5. Pulmonary Disease

Among the seven patients without bronchiectasis, the average diagnosis delay was 2.6 years, and LRIs occurred 2.1 times per year. However, among the five patients with bronchiectasis, the average diagnosis delay was 6.7 years, and LRIs occurred 9.6 times per year. Bronchiectasis was the most common HRCT finding; the other findings included enlarged hilar lymph nodes, nodules, and a mosaic pattern. All the patients with bronchiectasis had low B-cell percentages. In three of the patients, bronchiectasis was detected at the time of diagnosis. For another patient, bronchiectasis was detected two years after the diagnosis. All the patients with bronchiectasis were treated with inhaled corticosteroids, bronchodilators, and daily chest physiotherapy.

### 3.6. Autoimmune Disease

Five patients were diagnosed with autoimmune disease either initially or during follow-up. Two patients were diagnosed with Evans syndrome by pediatric hematology department, and CVID was considered after immune screening. Psoriasis, ankylosing spondylitis, JRA, and SLE were other autoimmune diseases detected among the patients. All patients diagnosed with autoimmune disease were over the age of 10 years. Absolute B-cell counts and IgM levels were also higher among autoimmune patients. Ig A levels were lower in patients with chronic enteropathy. No malignancy was seen in patients (Table 3).

### 3.7. Laboratory Findings

The median total serum IgG, IgM, and IgA levels were 605 ± 304, 87 ± 106, and 68 ± 41.9 mg/dL, respectively. While the IgG levels of 10 patients (83.3%) were initially low, the levels of two other patients were decreased at the follow-up (Table 4).

The peripheral blood lymphocyte subgroup was evaluated as normal (*n* = 3) in three patients. At diagnosis, low B-cell (*n* = 7) and CD4 (*n* = 5) levels were detected. In our study, the patients (*n* = 8) showed significantly decreased B-cell counts at the (approximately) five-year follow-up (*p* < 0.05). Five patients had normal B-cell levels at diagnosis, but at the follow-up their B-cell levels had decreased. Three patients with warts had low CD4+ T lymphocyte counts (Table 5).

## 4. Treatment

All the patients received IVIG replacement therapy at a dose of 500 mg/kg every three weeks. There were significant decreases in the LRI and gastroenteritis frequencies in the first year of IVIG treatment and a statistically significant decrease in the URI frequency at 5 years (*p* < 0.05). When the physical examination findings were evaluated after one and five years of IVIG replacement therapy, the patients with pathological sounds of lung at the time of diagnosis showed clear improvements (*p* < 0.05).

## 5. Discussion

CVID is one of the most frequently occurring types of primary hypogammaglobulinemia, and it is characterized by recurrent infections, autoimmunity, autoinflammatory, or hemophagocytosis syndromes. The symptoms may begin at any time of life, but they are seen more commonly at 5–10 and 20–30 years of age. There is no gender-based difference in the distribution [7]. In two studies, CVID was found to account for 20% of all PIDs, and the high rate of CVID patients was explained by the consanguinity frequency. In our study, CVID represented 1% of the PIDs, and this low proportion could be explained by the more advanced ages at symptom onset and the inability to diagnose patients with conditions other than frequent infections [8, 9]. In our study, the median patient age at disease onset was 7.2 ± 4.1 years, the mean diagnosis age was 11.6 ± 3.7 years, and the diagnosis delay was 4.3 ± 2.6 years. In the literature, the median disease onset age was nine months, the mean diagnosis ages were 10.5 and 7.8 years, and the diagnosis delay was 5.1 years [10–12].

Some researchers have suggested that CVID and a selective IgA deficiency may be inherited. Some individuals can have CVID, while other members of the same family have a selective IgA deficiency. Familial inheritance was detected in 25% of the cases with CVID and an IgA deficiency [13]. The rate of parental consanguinity was 75% (three or four degrees), and six patients had PID-related histories. The parents of all the bronchiectasis patients were in consanguineous marriages. Autosomal recessive mutations are thought to be common in Turkey, where consanguineous marriage is common, which suggests that the disease may be more serious in patients with recessive inheritance.

The most common symptoms among the patients were recurrent infections (URIs *n* = 8, LRIs *n* = 9, and gastroenteritis *n* = 5). In the Urschel et al. study (2009), the recurrent infection rates were 88%, 78%, and 34%, respectively [10]. Growth retardation was seen in 28% of the patients (*n* = 9, percentile of weight and height <3%). Therefore, children with growth retardation should be evaluated for immunodeficiencies.

Bronchiectasis is a structural abnormality characterized by the abnormal dilation and distortion of the bronchial tree, resulting in chronic obstructive lung disease. In our study, bronchiectasis was diagnosed in five patients. In the literature, the bronchiectasis rate was 24–54% in CVID patients [12–14]. Therefore, CVID patients should be closely monitored for lung infections and complications like bronchiectasis. In the current study, in the seven patients without bronchiectasis, the average diagnosis delay was 2.6 years, and LRIs occurred 2.1 times per year. However, in the five patients with bronchiectasis, the average diagnosis delay was 6.7 years, and LRIs occurred 9.6 times per year. A longer diagnostic delay, a lower B-cell percentage, and a higher number of LRIs were significant risk factors for bronchiectasis (*p* < 0.05). Bronchiectasis was the most common HRCT finding, and the other findings included enlarged hilar lymph nodes, nodules, and a mosaic pattern. An early diagnosis and treatment initiation are protective against CVID lung complications [15].

CVID patients can exhibit complaints of recurrent or persistent diarrhea, which may be due to an infection, superinfection, food allergy, or autoimmunity. Additionally, the incidences of Crohn's disease and ulcerative colitis were found to be higher among CVID-afflicted individuals than in the general population [12]. Five of the patients presented with recurrent gastroenteritis; however, no microorganisms were detected in the stool microscopy and culture. The stools were examined for sodium, osmolarity, reducing agents, occult blood, and alpha 1 antitrypsin and radiologically with normal results. After the IVIG treatment, the gastroenteritis frequency decreased.

Autoimmune disorders occur at higher incidence rates in CVID patients; for example, ITP and AIHA are reported frequently. Additionally, many forms of autoimmunity (JRA, pernicious anemia, autoimmune thyroiditis, alopecia areata, and SLE) have been described in CVID patients. Urschel et al. reported autoimmune diseases in 31% of the CVID patients. In our study, five patients presented with autoimmune diseases (cytopenia, Evans syndrome, psoriasis, ankylosing spondylitis, autoimmune neutropenia, JRA, and SLE). In accordance with the literature, the absolute B-cell counts, and IgM levels were higher than in the general population [5]. All the patients diagnosed with autoimmune diseases were over the age of 10 years. Patients diagnosed with CVID before the age of 10 years (i.e., early-onset CVID) are more susceptible to infections than noninfectious complications [16].

First apoptosis signal receptor (Fas) expression in the peripheral blood T and B cells and the Fas-mediated elimination of activated T cells were found to be elevated in seven children with Evans syndrome when compared with control children with acute ITP or other nonimmune disorders. It has also been shown that B cells from a subset of patients with CVID had increased Fas antigen expression and underwent enhanced apoptosis. In addition, Fas and Fas ligand system defects (either decreased or increased activity) can be seen in CVID and Evans syndrome. Initially, two patients were diagnosed with Evans syndrome by the pediatric hematology department, but the immune screening tests were compatible with CVID. Although we could not conduct genetic tests, after the laboratory and clinical evaluations, we considered those diagnosed with Evans syndrome to have CVID [2].

CVID patients are at a higher risk for neoplasms (i.e., hematological or solid tumors), and the most common type of malignancy is non-Hodgkin's lymphoma (2–8%). No malignancies developed in any of our patients; however, CVID patients must be monitored for autoimmunity, cytopenia, and malignancy [10].

Asthma is more common in CVID patients (40–50%) due to secondary inflammation. Generally, the total IgE and specific IgE levels and skin-prick test results are normal. Eight patients were treated for asthma with inhaled steroids before being diagnosed with CVID [17].

The occurrence of warts among CVID patients is uncommon, but T-cell deficiencies likely contribute to their susceptibility to warts. Having T lymphopenia and an impaired T-cell response predisposes these patients to disseminated viral infections (more specifically, herpes viruses and warts) [18, 19]. All the patients who presented with verruca and recurrent herpes labialis had lymphopenia and low CD4 levels; therefore, individuals with recurrent viral infections must be evaluated for CVID.

The IgG levels of 10 of the patients were low at the diagnosis, while the levels of another two patients were decreased in the follow-up. In all the patients, at least one of the specific antibody responses (isohemagglutinin and anti-HB) was found to be low. A single normal IgG level in patients who sustain frequent infections does not rule out a CVID diagnosis; therefore, the immunoglobulin levels and antibody responses should be checked and rechecked [13].

The number of B cells in CVID patients is variable. In the current study, 12% had no detectable B cells, 12% had reduced B cells, 54% had normal-range B cells, and 19% had increased B cells [20]. Eight patients with low B-cell numbers at the diagnosis were followed up for five years, and their B-cell counts were found to have progressively decreased. Initially, antigen-specific antibody responses and B-cell levels can be normal but decrease over time. Therefore, patients should be intermittently monitored for immunoglobulins and specific antibody responses. B-cell defects become prominent over time, which explains why CVID tends to occur in the second and third decades of life. Those patients who were complicated with bronchiectasis had low IgG and B lymphocyte levels. These are important parameters for identifying patients who are at risk of sustaining structural lung damage. In CVID patients, a lack of antigen-specific T cells is an important finding. Those patients with a low CD4+ T and low CD4/CD8 ratio are prone to opportunistic infections, splenomegaly, granulomatous disease, lymphoma, gastrointestinal disease, bronchiectasis, and autoimmune disease [21]. In our study, four patients with low CD4/CD8 ratios exhibited autoimmunity.

Nine of our patients' parents were blood relatives; therefore, we believe that, among our small study sample, autosomal recessive mutations could have led to the CVID occurrence. It is known that homozygous mutations in the TACI and ICOS genes result in reduced TACI and ICOS expression. To date, all the described ICOS mutations have been thought to be homozygous inherited, so we consider the possibility that the ICOS mutation rate was lower among our sample of patients. Homozygous and heterozygous mutations in TACI genes can trigger CVID, and heterozygous mutations are inherited as autosomal dominant. Given the consanguinity among the parents of many of our patients, we think that the TACI mutation frequency was lower among our patients. We believe that as-yet-undefined autosomal recessive inherited mutations may be responsible for the occurrence of CVID among many of the patients in our sample. For this reason, we are planning to undertake CVID genetic analyses in all of our patients.

## 6. Conclusion

CVID symptoms can be heterogeneous, and this condition can contribute to diagnosis delays. Moreover, CVID patients can be referred to any medical specialty (otolaryngology, respiratory medicine, gastroenterology, rheumatology, hematology, or oncology) according to their complaints. Therefore, all specialist physicians must remember CVID in the differential diagnosis of patients who present with recurrent or severe infections, autoimmunity, bronchiectasis, or malignancy. CVID management includes immunoglobulin replacement therapy, infection avoidance, pulmonary status monitoring, and evaluations for cytopenia and malignancy. An early diagnosis and suitable treatment can prevent mortality and morbidity in these cases.

## 7. Results and Recommendations

1. Among the CVID patients in our study, recurrent pulmonary infections were the most common manifestation.
2. CVID can present with autoimmunity, malignancy, hemophagocytic syndrome, bronchiectasis, and cytopenia, even in the absence of frequent infections.
3. The first severe infection without frequent infections should be investigated for immunodeficiency.
4. CVID patients should be followed for malignancy, autoimmune disease, and bronchiectasis.
5. Viral infections (such as recurrent herpes virus infections and verruca) are more common among patients with T-cell defects.
6. Pulmonary complications relate to the diagnosis delay, symptom onset age, and infection frequency prior to CVID treatment.
7. Primary immunodeficiencies are life-threatening in children. For an early diagnosis, a detailed anamnesis, family history, and physical examinations are very important. Laboratory support is also required. An early diagnosis and early treatment initiation will help preclude complications.

## Figures and Tables

**Table 1:** 

Patient	Sex	Diagnosis age (year)	Symptom of age (year)	Delay-diagnosis (year)	Consanguinity	History PID
1	M	15	13	2	Yes	Yes
2	F	7	5	2	Yes	No
3	F	13	7	6	Yes	No
4	F	4,5	3	1.5	Yes	Yes
5	M	10	10	0	No	Yes
6	M	12	3	9	Yes	Yes
7	M	18	13	5	Yes	No
8	M	10	3	7	Yes	Yes
9	F	10	4	6	Yes	Yes
10	M	17,3	15	2,3	No	No
11	M	11	6	5	No	No
12	F	11,7	5	6,7	Yes	No

**Table 2:** 

Patient	Growth retardation	Lenfadenopathy	Organomegaly	Warts	Clubbing finger	Recurrent herpes labialis	Abnormal lung auscultation
1	Yes	No	Yes	No	No	No	No
2	Yes	No	No	No	No	No	No
3	Yes	No	No	No	No	No	Crackles
4	Yes	No	No	No	No	No	No
5	Yes	No	No	Yes	No	Yes	No
6	Yes	No	No	Yes	Yes	Yes	Crackles
7	Yes	No	No	No	Yes	No	Crackles
8	Yes	No	No	Yes	Yes	No	Crackles
9	Yes	No	Yes	No	No	No	No
10	No	Yes	Yes	No	No	No	No
11	No	No	Yes	Yes	No	Yes	No
12	No	No	No	Yes	No	No	Crackles

**Table 3:** 

Patient	Clinical presentation	Recurrent gastroenteritis	Tuberculosis	Autoimmune disease	Bronchiectasis	Asthma
1	Recurrent URTI-LRTI	No	No	Ankylosing-spondylitis-Psoriasis	No	Yes
2	Recurrent URTI-LRTI	Yes	No	No	No	Yes
3	Recurrent URTI-LRTI	Yes	No	No	Yes	Yes
4	Recurrent URTI-LRTI	No	No	No	No	No
5	Empyema	No	Yes	No	No	No
6	Recurrent URTI-LRTI	No	No	No	Yes	Yes
7	Recurrent LRTI	Yes	Yes	No	Yes	Yes
8	Recurrent URTI-LRTI	Yes	Yes	No	Yes	Yes
9	Recurrent URTI-LRTI	Yes	No	Rheumatoid arthritis	No	Yes
10	Thrombocytopenia-anemia	No	No	Evans syndrome	No	No
11	Thrombocytopenia-anemia	No	No	Evans syndrome	No	No
12	Recurrent URTI-LRTI	No	No	SLE	Yes	Yes

**Table 4:** 

Patient	L	N	T	Lymphocyte count (mm3)	IgG (mg/dL)	IgA (mg/dL)	IgM (mg/dL)	Ig E	Anti-Hbs (U/L)	Isohemagglutinin
1	No	No	No	1140	452 (L)	82 (L)	100 (N)	20	>10	1/8
2	Yes	No	No	2500	1110 (N)	6,6 (L)	39 (L)	5	Negative	1/2
3	Yes	No	No	3240	1250 (N)	396 (N)	41.8 (L)	23	>10	1/2
4	Yes	Yes	No	1800	447 (L)	126 (N)	36 (L)	107	Negative	1/2
5	No	Yes	Yes	12.600	653 (L)	29 (L)	55 (N)	20	Negative	1/4
6	Yes	No	No	2150	156 (L)	6 (L)	54 (N)	19	Negative	1/2
7	Yes	No	No	1000	558 (L)	23 (L)	18 (L)	14	>10	1/4
8	Yes	No	No	1700	277 (L)	23 (L)	19 (L)	14	Negative	1/2
9	Yes	No	No	1400	690 (L)	190 (N)	75 (N)	14	Negative	1/4
10	No	Yes	No	935	351 (L)	36 (L)	80 (L)	5	Negative	1/4
11	Yes	No	No	4770	620 (L)	46 (L)	113 (N)	5	>10	1/8
12	Yes	No	No	1150	700 (L)	82 (N)	163 (N)	5	Negative	1/4

L: temporary lymphopenia; N: temporary neutropenia; T: temporary thrombocytopenia; N: normal range; L: low.

**Table 5:** 

P	CD19 (%)	Range (%)	CD3 (%)	Range (%)	CD4 (%)	Range (%)	CD4/*µ*L	CD8 (%)	Range (%)	CD4/CD8
1	12 (N)	(10–30)	69 (N)	(58–82)	42 (N)	(26–48)	478	29 (N)	(16–32)	1.4
2	5 (L)	(10–27)	83 (N)	(57–81)	37 (N)	(24–47)	925	41 (N)	(17–37)	0.9
3	8 (L)	(10–30)	72 (N)	(58–82)	41 (N)	(26–48)	1328	31 (N)	(16–32)	1.3
4	6 (L)	(11–31)	85 (N)	(55–79)	43 (N)	(26–49)	774	36 (N)	(9–35)	1.1
5	8 (L)	(10–27)	90 (N)	(57–81)	11 (L)	(24–47)	1386	75 (H)	(17–37)	0.1
6	7 (L)	(10–30)	89 (N)	(58–82)	21 (L)	(26–48)	336	54 (H)	(17–37)	0.38
7	6 (L)	(10–30)	82 (N)	(58–82)	27 (N)	(26–48)	270	45 (H)	(17–37)	0.6
8	9 (L)	(10–30)	70 (N)	(58–82)	33 (L)	(26–48)	560	36 (N)	(17–37)	0.9
9	12 (N)	(10–30)	90 (N)	(58–82)	30 (N)	(26–48)	420	37 (N)	(17–37)	0.8
10	10 (N)	(10–30)	76 (N)	(58–82)	30 (N)	(26–48)	280	40 (H)	(17–37)	0.75
11	26 (N)	(10–30)	53 (L)	(58–82)	24 (L)	(26–48)	900	28 (N)	(17–37)	0.8
12	8 (L)	(10–30)	49 (L)	(58–82)	20 (L)	(26–48)	310	28 (N)	(17–37)	0.7

## Data Availability

Please contact Gülsüm Alkan for data requests.

## Abbreviations

AGE: Acute gastroenteritis
AIHA: Autoimmune hemolytic anemia
ESID: European Society of Immunodeficiency Diseases
HRCT: High-resolution computed tomography
ICOS: Inducible costimulator
ITP: Idiopathic thrombocytopenic purpura
IVIG: Intravenous immunoglobulin
JRA: Juvenile rheumatoid arthritis
LRI: Lower respiratory infection
LRIs: Lower respiratory infections
PID: Primary immunodeficiency
SLE: Systemic lupus erythematosus
TACI: Calcium-modulator and cyclophilin ligand interactor
URT: Upper respiratory infection
URTs: Upper respiratory infections.
